# Differentiating Progressive Supranuclear Palsy and Corticobasal Syndrome: Insights from Cerebrospinal Fluid Biomarkers—A Narrative Review

**DOI:** 10.3390/medicina61040701

**Published:** 2025-04-11

**Authors:** Alexandros Giannakis, Spiridon Konitsiotis, Chrissa Sioka

**Affiliations:** 1 Department of Neurology, Faculty of Medicine, School of Health Sciences, University of Ioannina, Stavrou Niarchou Av., University Campus, 45500 Ioannina, Greece; papadates@gmail.com (A.G.);; 2 Department of Nuclear Medicine, Faculty of Medicine, School of Health Sciences, University of Ioannina, Stavrou Niarchou Av., University Campus, 45500 Ioannina, Greece

**Keywords:** progressive supranuclear palsy, corticobasal syndrome, biomarker, differential diagnosis, cerebrospinal fluid

## Abstract

*Background and Objectives*: Despite ongoing research and evolving diagnostic criteria, progressive supranuclear palsy (PSP) and corticobasal syndrome (CBS) remain notoriously difficult to differentiate, largely due to their overlapping clinical presentations and the absence of definitive biomarkers. *Materials and Methods*: We provide a comprehensive review of cerebrospinal fluid (CSF) biomarkers, which have proven valuable in the diagnosis of other neurodegenerative conditions, and their application to PSP and CBS. *Results*: The most promising results derive from a combination of biomarkers associated with Parkinson’s disease, Alzheimer’s disease, and neurofilament light chain. Furthermore, CSF proteomics analysis offers valuable insights into the pathogenesis of PSP and CBS and could also contribute to accurate diagnosis. *Conclusions*: CSF biomarkers hold significant potential for improving the differential diagnosis of PSP and CBS. A stepwise combination approach—starting with CSF α-synuclein and neurofilament light chain, followed by amyloid-β42 and total and phosphorylated tau—may provide clinicians with a practical framework for distinguishing PSP and CBS from other neurodegenerative disorders. To advance this field, future efforts should prioritize large-scale, multicenter studies employing standardized methodologies to enhance the validity and reproducibility of biomarker-based diagnostics. Importantly, considering the frequent pathological overlap between PSP and CBS, future studies would greatly benefit from pathology-confirmed cohorts to ensure diagnostic accuracy and to better delineate biomarker profiles across these challenging conditions.

## 1. Introduction

Progressive supranuclear palsy (PSP) and corticobasal syndrome (CBS) present significant diagnostic challenges due to their overlapping clinical features [1,2]. Even for experienced neurologists, differentiating between these atypical parkinsonian disorders (APDs) can be exceedingly difficult [3,4].

While current diagnostic criteria for PSP and CBS have improved sensitivity and specificity, they often rely on complex combinations of clinical features, pushing the limits of clinical assessment [1,2]. Consequently, a substantial proportion of patients receive inaccurate diagnoses during their lifetime, with definitive confirmation only possible through post-mortem examination [5]. For instance, autopsy studies reveal that only 38% of patients clinically diagnosed with CBS are confirmed to have corticobasal degeneration (CBD) [6]. Notably, PSP is a common underlying pathology in patients presenting with CBS (24%), highlighting the diagnostic overlap. Other neurodegenerative diseases, such as Alzheimer’s disease (AD), also contribute to the diagnostic complexity (15%) [6]. Compounding the issue, both PSP and CBS diagnostic criteria include subtypes that mirror each other, blurring the lines further [1,2]. Moreover, emerging perspectives place CBS and PSP within broader and more diverse neurodegenerative spectra, such as frontotemporal dementia (FTD) and posterior cortical atrophy (PCA), further complicating clinical diagnosis [7,8]. This diagnostic uncertainty generates anxiety for patients, who seek answers about their condition. Furthermore, the inability to accurately distinguish between PSP and CBS in vivo hinders the development of tailored therapeutic protocols, leaving patients with largely symptomatic treatments of limited efficacy [9].

Interestingly, PSP and CBS criteria rely on clinical characteristics, without incorporating specific biomarkers as either core or supportive features [1,2]. This contrasts with the diagnostic approaches for other neurodegenerative disorders, where the complexity of clinical presentations necessitates the use of biomarkers to mitigate the risk of misdiagnosis [10]. AD provides a prime example; initially, cerebrospinal fluid (CSF), blood, and imaging biomarkers served as adjuncts to clinical evaluation [11], but their significance has grown substantially with the adoption of a biological definition of the disease [12]. In the most recent criteria, they now constitute a sufficient and necessary condition for diagnosis [13]. Similarly, the diagnostic criteria for dementia with Lewy bodies (DLBs) include several biomarkers as supportive diagnostic features, such as fludeoxyglucose-18 (FDG) positron emission tomography (PET) and dopamine transporter imaging with single-photon emission computed tomography (DaT-SPECT) [14]. Notably, a normally functioning presynaptic dopaminergic system on DaT-SPECT serves as an absolute exclusion criterion for Parkinson’s disease (PD) [15]. Furthermore, the recently released European recommendations for diagnosing neurocognitive disorders propose a biomarker-based diagnostic algorithm—utilizing CSF, blood, and imaging biomarkers—for neurodegenerative diseases, including PSP and CBS [16].

The purpose of this paper is to present a comprehensive narrative review of diagnostic tools related to CSF potential biomarkers for the differential diagnosis of PSP and CBS. This review aims to explore how these biomarkers can not only help distinguish PSP from CBS but also differentiate both conditions from other neurocognitive disorders, thereby enhancing diagnostic accuracy and guiding clinical decision-making.

## 2. Cerebrospinal Fluid Biomarkers

### 2.1. Tau Protein

PSP and CBS share a common pathological hallmark: the aggregation of four-repeat (4R) tau protein [17]. Normally, tau protein is crucial for maintaining the integrity of the neuronal cytoskeleton by binding to tubulin and stabilizing microtubules [18]. The MAPT gene encodes tau protein, and alternative splicing of this gene yields six distinct tau isoforms [19]. Each isoform contains microtubule-binding domains (MTBDs). Based on the number of MTBD repeats in the tau molecule, tau is classified as either three-repeat (3R) or four-repeat (4R), while several other phosphorylated forms exist [19,20]. While tau protein possesses numerous phosphorylation sites, hyperphosphorylation (p-tau) disrupts its microtubule-binding capacity, resulting in cytoskeletal instability and insolubility, which ultimately promotes the formation of cytoplasmic inclusions [20]. PSP and CBS are classified as primary 4R tauopathies, characterized by cytoplasmic inclusions of 4R tau protein, alongside argyrophilic grain disease (AGD) and globular glial tauopathy [21]. Conversely, AD is characterized by intracellular neurofibrillary tangles composed of a mixture of 3R and 4R tau protein aggregates, which form secondarily to extracellular amyloid-beta (Aβ) plaque deposition [22]. Similarly, in other primary tauopathies, such as Pick’s disease, tau aggregations are predominantly 3R [23].

Table 1 summarizes the main findings of CSF tau protein studies.

A study investigating CSF tau levels in PSP showed that total tau (t-tau) and p-tau levels are typically within the normal range in both the PSP-Richardson’s syndrome (PSP-RS) and PSP with predominant parkinsonism (PSP-P) subtypes, the two most common variants. Furthermore, no significant differences in tau levels were observed between these subtypes [24]. Borroni and co-authors were the first to compare CSF tau levels between PSP and other neurodegenerative diseases. Specifically, they employed a semiquantitative immunoprecipitation method to analyze the ratio of a truncated (33 kDa) to an extended (55 kDa) tau protein isoform [25]. Their findings revealed a significantly reduced 33 kDa/55 kDa ratio in PSP patients compared to those with CBD, PD, DLB, AD, and FTD and age-matched healthy controls. Notably, this ratio reduction correlated with brainstem atrophy, as assessed by magnetic resonance imaging (MRI) voxel-based morphometry (VBM) in PSP patients [25]. Furthermore, Borroni and co-authors subsequently developed a diagnostic algorithm that integrated the CSF tau ratio with midbrain-to-pons atrophy, achieving a high sensitivity of 94.2% and a specificity of 84.0% in differentiating PSP from CBD and FTD [26]. However, Kuiperij and co-authors were unable to replicate these findings in a subsequent study [27].

CSF tau has also been investigated for differentiating PSP from other forms of FTD. Recent findings by Heikkinen and co-authors revealed that t-tau and p-tau CSF levels were significantly lower in PSP patients compared to those with behavioral variant frontotemporal dementia (bvFTD) [8]. Notably, they also observed a significant overlap in diagnostic criteria among PSP, CBS, and bvFTD patients, even within a tertiary neurology clinic setting [8]. Furthermore, Saijo and co-authors employed real-time quaking-induced conversion (RT-QuIC) assays on post-mortem CSF and crude brain homogenates from patients with diverse neurodegenerative conditions, including various forms of FTD, such as PSP, CBD, Pick’s disease, amyotrophic lateral sclerosis (ALS), frontotemporal lobar degeneration with TDP-43 (FTLD-TDP), and frontotemporal dementia with parkinsonism linked to chromosome 17 (FTDP-17) [28]. They also included patients with AD, chronic traumatic encephalopathy (CTE), DLB, cerebrovascular disease, and chronic inflammatory demyelinating polyneuropathy. Utilizing a 3R tau fragment as a substrate, they achieved high sensitivity and specificity in differentiating the 3R-related Pick’s disease from 4R tauopathies (PSP, CBD, and AGD) and mixed 3R/4R tauopathies (AD and CTE). [28]. Subsequently, utilizing a 4R tau substrate, they conducted RT-QuIC assays on post-mortem crude brain homogenates and CSF samples from patients with PSP, CBD, Pick’s disease, ALS, FTLD-TDP, FTDP-17, AD, PD, DLB, multiple system atrophy (MSA), central nervous system (CNS) lymphoma, and meningeal carcinomatosis [29]. Tau seeds were detected in all pathologically confirmed PSP and CBD cases, but not in the other diagnostic groups. However, the CSF samples from the living patients exhibited weaker seeding activities, although assay responses were significantly higher in the patients with clinically diagnosed PSP or CBS [29].

The application of CSF tau biomarkers used in AD diagnosis has been extended to PSP. Using multiplex immunoassay, researchers found that AD patients had significantly higher levels of t-tau and tau phosphorylated at threonine 181 (p-tau181) in their CSF compared to both PSP patients and healthy controls [30]. In contrast, ELISA analysis demonstrated that PSP patients exhibited lower CSF concentrations of N-terminal and C-terminal tau than both AD patients and controls [30]. Furthermore, CSF levels of another tau fragment, N-224, were significantly elevated in AD patients compared to individuals with mild cognitive impairment (MCI), subjective cognitive decline (SCD), and other neurodegenerative diseases, including PSP, PD, and MSA [31]. Additionally, two 4R tau species containing MTBDs in the CSF could differentiate AD from other neurodegenerative diseases (PSP, CBD, Pick’s disease, and AGD) but do not distinguish among these non-AD conditions [32]. Finally, utilizing both CSF p-tau181 and 18F-PI-2620 tau-PET imaging, researchers distinguished AD from 4R tauopathies (PSP or CBS) [33]. AD was associated with elevated CSF p-tau181 and cortical 18F-PI-2620 binding, whereas PSP and CBS exhibited predominantly subcortical 18F-PI-2620 binding [33]. As will be discussed later, in AD, the CSF Aβ42/Aβ40 ratio is decreased due to a specific reduction in Aβ42, while Aβ40 levels remain relatively unchanged [35]. Conversely, both Aβ42 and Aβ40 are diminished in the CSF of PSP patients, irrespective of cerebral amyloid burden, resulting in an Aβ42/Aβ40 ratio closer to healthy controls [35,36]. Finally, it is important to note that phosphorylated tau at threonine 217 (p-tau217) is a highly sensitive and specific biomarker for early-stage AD, now included in the new diagnostic criteria [13]. When measured in CSF alongside the Aβ42/Aβ40 ratio and the p-tau217/t-tau217 ratio, it can effectively differentiate AD from PSP, CBS, and bvFTD. AD is characterized by a high p-tau217/t-tau217 ratio and a low Aβ42/Aβ40 ratio, whereas the other conditions exhibit a low p-tau217/t-tau217 ratio and a normal Aβ42/Aβ40 ratio. This biomarker combination can also detect a mixed 3R/4R tauopathy—similar to AD but without Aβ deposition—resulting from the rare MAPT R406W mutation [34].

### 2.2. Amyloid Beta

Amyloid plaques are the defining pathological feature of AD [12]. These plaques are primarily composed of Aβ peptides, which originate from the amyloid precursor protein (APP), a protein embedded in cell membranes [37,38]. The processing of APP is primarily mediated by two enzymes: α-secretase and β-secretase. α-secretase cleavage of APP produces the soluble APP-α fragment. In contrast, β-secretase cleavage results in the APP-β fragment, which is insoluble and prone to aggregation in the extracellular space [37,38]. These insoluble Aβ fragments are further classified based on their C-terminal structure. Specifically, Aβ42 is the dominant form found in extracellular amyloid plaques, while Aβ40 tends to accumulate within the vascular endothelium, contributing to cerebral amyloid angiopathy [37,38].

CSF levels of Aβ42, t-tau, and p-tau181 were quantified using ELISA in a large cohort of patients with dementia [39]. In the patients with pathologically confirmed AD, this biomarker profile correctly identified 92% of cases. However, a notable proportion of the patients with other dementias, including CBD, DLB, FTD, and vascular dementia (VaD), also exhibited an AD biomarker profile. Conversely, the patients with PSP demonstrated normal Aβ42 and tau values in 90% of cases [39]. In the first study to examine AD biomarkers specifically in parkinsonian syndromes, Bech and co-authors found that CSF Aβ42 levels were particularly low in DLB patients [40]. Additionally, soluble APP-α and APP-β fragments in the CSF were lower in DLB and MSA compared to PSP and PD [40]. Interestingly, in a meta-analysis of 16 eligible studies encompassing 1684 participants, researchers compared CSF levels of soluble APP-α and APP-β across various neurodegenerative diseases and cognitive disorders [41]. They found that APP-β levels were significantly higher in AD and PD than in PSP and CBS and were also elevated in MCI that progressed to AD compared to PSP [41]. Similarly, Nutu and co-authors, using CSF levels of Aβ15 and Aβ16, sought to discriminate between patients with PD, PD dementia (PDD), AD, DLB, MSA, CBD, and PSP [42]. They reported significantly lower values for these Aβ fragments in PSP, PD, PDD, and MSA [42]. Additionally, the combination of neurocognitive tests and CSF AD biomarkers demonstrated 100% sensitivity but 75% specificity in distinguishing AD from a range of other neurodegenerative diseases, such as PSP, CBS, DLB, FTD, primary progressive aphasia (PPA), and PCA [43]. Even so, the CSF Aβ42/Aβ40 ratio, t-tau, and p-tau181 successfully differentiated AD from various FTD variants in patients with intermediate to high AD neuropathologic changes [35]. Notably, t-tau levels were reduced in PSP patients relative to other FTD variants, while FTLD-TDP had a reduced p-tau/t-tau ratio [35]. In another autopsy-confirmed study, the combination of CSF Aβ42 and p-tau181 levels was found to predict AD pathology and exclude other neurocognitive syndromes, such as PSP, FTD, and PPA [44]. Interestingly, Paraskevas and co-authors reported that 15.3% of patients with CSF biomarkers consistent with AD displayed clinical presentations beyond typical amnestic AD, encompassing PSP-RS, CBS, PPA, and NPH [45]. The study’s reliance on a tertiary academic center population raises the possibility of selection bias. Nevertheless, these results challenge conventional understanding by suggesting that atypical clinical manifestations of AD pathology, including PSP and CBS, may be more frequent than previously appreciated [45].

Although extensive research has explored the use of CSF AD biomarkers to discriminate AD from other neurodegenerative diseases, studies focusing on PSP remain scarce. A study examined CSF levels of Aβ42, t-tau, and p-tau and the ratios of p-tau/t-tau and Aβ42/p-tau in patients with PSP, PD, and healthy controls [46]. The results indicated that a CSF Aβ42 cutoff value of 623 pg/mL effectively differentiated PSP from PD and that lower Aβ42 levels were associated with greater PSP disease severity [46]. Additionally, a p-tau/t-tau ratio cutoff of 0.185 was found to aid in the differential diagnosis of PSP and PD [46]. Intriguingly, a patient presenting with clinical PSP, CSF findings indicative of AD, and negative amyloid PET imaging was ultimately diagnosed post-mortem with typical pathological changes of AD [47].

CSF AD biomarkers have been more thoroughly investigated in CBS. Borroni and co-authors found that 16.6% of patients with a CBS phenotype exhibited CSF biomarkers consistent with AD [48]. This subgroup also displayed early memory impairment [48]. In a similar vein, a separate cohort study reported that 18% of CBS patients demonstrated CSF profiles consistent with AD. This subgroup was mainly characterized by Gerstmann syndrome, while patients with CSF inconsistent with AD commonly displayed prefrontal and/or semantic/logopenic deficits [49]. Constantinides and co-authors reported an even higher proportion of CBS patients with CSF biomarkers consistent with AD. This subgroup was characterized by older age, later disease onset, and a greater prevalence of alien-limb phenomena, a hallmark of CBS [50]. However, the same study group revealed that different interpretation criteria for CSF AD biomarkers lead to substantial variability in determining AD pathology in CBS [51]. Furthermore, a separate study group reported that only 5% of CBS patients exhibited CSF biomarkers consistent with AD [52]. Finally, Garcia-Cordero and co-authors categorized patients with CBS or PSP based on their CSF AD biomarker status. The biomarker-positive group exhibited poorer neurocognitive performance and distinct brain atrophy patterns compared to the biomarker-negative group [53].

### 2.3. Neurofilament Light Chain

Neurofilament light chain (NfL) is a structural protein constituent of the neurofilament complex within large-caliber myelinated axons, working in conjunction with neurofilament heavy and medium chain subunits [54]. The neurofilament complex functions as an axonal cytoskeletal network, providing essential structural support [55,56]. In physiological aging, basal levels of NfL are released into the interstitial fluid, CSF, and circulation [55,56]. Conversely, significant axonal disruption, occurring in conditions such as stroke, traumatic brain injury, and neurodegenerative disorders, results in substantial release of NfL into the CSF and blood, rendering it a sensitive, yet nonspecific, biomarker for axonal injury [54]. Table 2 summarizes the main findings of CSF NfL protein studies.

Extensive research has examined CSF NfL in parkinsonian disorders. Holmberg and co-authors, employing ELISA, compared CSF NfL levels among patients with PD, PSP, and MSA. Their findings revealed significantly elevated NfL levels in the PSP and MSA groups relative to the PD group [57]. Correspondingly, CSF neurofilament heavy chain was found to be significantly increased in PSP and MSA relative to PD. Although it effectively differentiated PSP from PD with high specificity (94.4%), its moderate sensitivity (76.5%) suggested limitations in its use as a sole diagnostic marker [58]. A more recent study found significantly higher CSF NfL levels in PSP, CBD, and MSA patients compared to PD patients and healthy controls. Furthermore, NfL levels correlated with midbrain atrophy in PSP and localized atrophy across the midbrain and pons in CBD [59]. Additionally, CSF NfL has shown promise in distinguishing patients with PSP from healthy controls and α-synucleinopathies, specifically PD and DLB [60].

Furthermore, CSF NfL has been investigated across various FTD syndromes and diverse neurodegenerative cohorts. A study employing ELISA compared CSF NfL levels in patients with PSP, CBS, bvFTD, PPA, AD, and PD and healthy controls. The results showed elevated NfL levels in bvFTD and PPA relative to all other groups [61]. A cohort study with a broad range of neurodegenerative diseases, including PSP, CBS, PD, PD with MCI, PDD, DLB, FTD, ALS, AD, and MCI, and healthy controls used ELISA to compare CSF NfL levels. ALS and FTD exhibited the highest NfL levels. Furthermore, among parkinsonian syndromes, PSP and CBS showed the highest levels [62].

A comprehensive meta-analysis, incorporating 8 studies and a total of 341 patients with PD and 396 patients with APDs, namely, PSP, CBD, and MSA, was performed to determine the diagnostic performance of CSF NfL in differentiating PD from APDs [63]. The pooled sensitivity and specificity were determined to be 82% and 85%, respectively. However, subgroup analyses demonstrated that variations in study design significantly affected both sensitivity and specificity [63]. A final, more extensive systematic review and meta-analysis, incorporating 36 studies, evaluated CSF NfL levels across a broad spectrum of neurodegenerative diseases [64]. The highest observed increases in CSF NfL concentrations were in ALS, Creutzfeldt–Jakob disease, and Huntington’s disease [64]. In contrast, CSF NfL levels were consistently within normal limits in PD, while significant elevations were noted in PSP and MSA, as previously displayed [57,64].

### 2.4. α-Synuclein

PD and DLB are characterized by Lewy bodies, i.e., intracytoplasmic neuronal inclusions of misfolded α-synuclein [65,66]. MSA also features similar α-synuclein inclusions but within oligodendrocytes, leading to the classification of these three disorders as synucleinopathies [67,68]. α-synuclein is primarily concentrated in presynaptic terminals, where it participates in a range of biological functions [69]. Although its exact role is not fully elucidated, studies suggest its involvement in presynaptic vesicle exocytosis, modulating striatal network plasticity, and regulating astrocyte glutamatergic signaling [69]. The process by which α-synuclein forms oligomers and subsequently aggregates into Lewy bodies remains a subject of ongoing research [70].

The emerging role of α-synuclein as a biomarker for synucleinopathies has also been explored in the differential diagnosis of APDs, including PSP and CBS. Tokuda and co-authors conducted a study to evaluate the potential of α-synuclein oligomers in CSF as a biomarker for PD. Using ELISA, they measured the levels of α-synuclein oligomers and calculated the ratio of oligomers to total α-synuclein [71]. In a comparison between PD patients and control subjects, the oligomers/total-α-synuclein ratio was found to be significantly elevated in the PD patients [71]. This ratio demonstrated a high sensitivity of 89.3% and a specificity of 90.6% for distinguishing PD from the controls. Additionally, a subsequent cross-sectional pilot study revealed that CSF oligomer levels were also significantly higher in the PD patients compared to those with PSP [71]. Mondello and co-authors investigated α-synuclein and ubiquitin carboxy-terminal hydrolase L1 (UCH-L1) levels in the CSF of patients with PD, MSA, PSP, and CBD [72]. They observed that α-synuclein levels were significantly lower in synucleinopathies (PD and MSA) compared to tauopathies (PSP and CBD) [72]. Furthermore, UCH-L1 levels, a component of the ubiquitin–proteasome system, which is implicated in PD, were significantly decreased in the PD patients compared to both the APD patients and controls. UCH-L1 levels were also significantly decreased in the PSP patients compared to the controls, but not in CBD [72]. However, a subsequent, comprehensive study involving PD, PSP, CBD, DLB, MSA, and vascular parkinsonism patients showed no significant variation in CSF α-synuclein concentrations [73]. A meta-analysis comprising 12 studies, with a total of 1131 patients diagnosed with PD, PSP, DLB, and MSA, examined CSF α-synuclein concentrations. Among these, the three studies that specifically compared PD and PSP (517 PD and 92 PSP patients) showed a marginally lower α-synuclein concentration in PD patients [74].

The introduction of a seed amplification assay (SAA) using RT-QuIC for the detection of even minute levels of aggregated α-synuclein in biological tissues has revolutionized the field of parkinsonian syndrome biomarkers [75]. Manne and co-authors utilized monomeric recombinant human wild-type α-synuclein as a substrate to detect α-synuclein aggregates in CSF samples from PD and PSP patients, as well as post-mortem brain tissues from PD, DLB, and AD patients and healthy controls [76]. The protein aggregation rate (PAR) was significantly elevated in PD and DLB brain tissues compared to AD and the controls, demonstrating high reproducibility, sensitivity (94%), and specificity (100%) [76]. Similarly, the CSF samples from the PD patients exhibited significantly higher PAR values compared to the controls, with 100% sensitivity and specificity [76]. While two of the five PSP CSF samples showed increased PAR values, suggesting potential Lewy body pathology, these values did not significantly differ from the controls [76]. Similarly, Vaughan and co-authors utilized SAA to examine α-synuclein aggregates in CSF samples from patients with PSP and CBS, aiming to investigate the clinicopathological features of α-synuclein in these disorders [77]. The study revealed that one PSP and two CBS cases were positive for MSA-type SAA. Additionally, 10.2% of the PSP patients tested positive for PD-type SAA, compared to 25.7% of the CBS patients. Furthermore, the finding that the PSP patients were older and had a shorter disease duration suggested a potential relationship between α-synuclein and the clinical course of PSP [77]. Lastly, a comprehensive systematic review and network meta-analysis were conducted to compare the relative accuracy of SAA in various biological samples [78]. When focusing on CSF, the pooled sensitivity and specificity were 0.92 and 0.84, respectively, for distinguishing PD from PSP [78]. In a separate comparison, the pooled sensitivity and specificity for CSF were 0.94 and 0.95, respectively, when differentiating PD from CBD [78].

### 2.5. Inflammatory Biomarkers

Inflammatory pathways and their contribution to neuroinflammation are actively being investigated as key factors in neurodegenerative disease pathogenesis. Hall and co-authors analyzed a broad panel of inflammatory biomarkers in the CSF of patients with PSP, PD, PDD, and MSA and healthy controls [79]. Their findings indicated lower chitinase-3-like-1 (YKL-40)—a glycoprotein that is upregulated under inflammatory conditions—concentrations in PSP compared to the controls, reduced monocyte chemotactic protein-1 (MCP-1)—a chemokine that is associated with microglial activation—in PDD relative to PSP, and a positive correlation between interleukin 6 IL-6 concentration and motor disability specifically in PSP [79]. In a study comparing microglia-derived cytokines in CSF, Starhof and co-authors found that tumor necrosis factor α (TNF-α), interleukin 1β (IL-1β), and IL-6 were significantly increased in PSP and MSA compared to PD but not when compared to controls [80]. Ayton and co-authors examined 11 acute-phase proteins in the CSF of patients with PSP, PD, PDD, DLB, and MSA [81]. They reported that haptoglobin was selectively increased in PSP [81]. Furthermore, they demonstrated that a combination of haptoglobin, ferritin, and transthyretin could effectively distinguish PSP from MSA, with ferritin and transthyretin showing selective increases in MSA [81]. Takahashi and co-authors measured diacron-reactive oxygen metabolites and biological antioxidant potential in the CSF of patients with PSP and PD and healthy controls [82]. They observed a significantly elevated antioxidant capacity in the PSP patients when compared to the PD patients [82]. Kynurenic acid, a tryptophan metabolite produced by astrocytes and linked to inflammatory pathways, shows elevated levels in AD relative to PSP, FTD, and ALS [83]. On the other hand, Jabbari and co-authors measured NfL and an inflammatory biomarker panel in patients with PSP, CBS, MSA, and PD and healthy controls [84]. NfL was more effective than the inflammatory biomarker panel in distinguishing between PD and APDs [84].

CSF inflammatory biomarkers have also been the subject of comparative studies between PSP and other neurodegenerative diseases. An immunoblot analysis of nitrated tyrosine residue-containing proteins in CSF revealed that manganese superoxide dismutase was significantly increased in ALS, increased in PSP, and only slightly increased in PD and AD [85]. A study examined CSF levels of glial inflammatory markers YKL-40, chitotriosidase 1 (CHIT1), and glial fibrillary acidic protein (GFAP) in patients with prionopathies, AD, and FTLD. The FTLD group included a tauopathy subgroup comprising PSP and CBS [86]. Compared to controls, all markers were significantly increased in each disease group. YKL-40 levels were highest in prion diseases compared to AD and FTLD [86]. Within FTLD, the ALS subgroup had higher YKL-40 levels than other FTLD subgroups, and CHIT1 levels showed significant variation between FTLD-TDP tauopathy subgroups [86]. CSF YKL-40 levels, as measured by ELISA in an older study, were also increased in very mild and mild AD-type dementia compared to PSP and controls [87]. Furthermore, leucine-rich α2-glycoprotein, an inflammatory-induced protein, exhibited elevated levels in the CSF of patients with PSP and PDD relative to those with AD and NPH and healthy controls [88].

### 2.6. Proteomics

CSF proteomics has also been explored in PSP. Marques and co-authors conducted shotgun proteomics analysis of tryptic peptides in the CSF of patients with PD, MSA, PSP, and non-neurological controls [89]. Their study identified 191 significantly different tryptic peptides. Subsequently, a selected reaction monitoring assay was performed on 34 selected peptides, revealing that 14 of these peptides effectively differentiated PD from PSP and MSA, demonstrating moderate-to-high accuracy [89]. Constantinescu and co-authors employed surface-enhanced laser desorption/ionization time-of-flight mass spectrometry to analyze CSF samples [90]. Their study identified four CSF proteins—ubiquitin, β2-microglobulin, and two fragments of secretogranin—that could differentiate PD from PSP, CBD, and MSA [90]. However, importantly, these protein changes were not specific to any particular subgroup of APDs, likely reflecting the widespread neurodegeneration common to all APDs [90]. Recently, Mravinacova and co-authors utilized antibody-based suspension bead array technology to measure 69 CSF proteins in PSP, CBS, AD, bvFTD, PPA, ALS, and controls [91]. No disease-specific protein associations were found, except for neurofilament medium chain and myelin basic protein in ALS and neurogranin in AD. Notably, significant inter-individual variability was observed [91]. In a study aimed at identifying diagnostic biomarkers for PD and APDs, researchers employed a multiplex proximity extension assay to analyze CSF proteins in patients with PSP, CBS, PD, and MSA and in controls [92]. The analysis revealed that midkine and DOPA decarboxylase were significantly increased in the CSF of the PD patients, but not in the APD patients or the controls. Of particular interest, Wnt inhibitory factor 1 demonstrated a consistent downregulation specifically in PSP and CBS [92].

Magdalinou and co-authors employed a proteomic approach, utilizing tryptic digestion, liquid chromatography–mass spectrometry, and isobaric labeling, to identify and quantify proteins in CSF [93]. This method revealed 79 tryptic peptides from 26 proteins that showed significant differences in CSF levels between PD patients and those with PSP, CBS, and MSA [93]. These proteins were primarily associated with acute phase responses, inflammation, and synaptic function, all of which play critical roles in neurodegenerative processes [93]. Interestingly, Jang and co-authors recently performed a CSF proteomic analysis using tandem mass tag-based quantification, a quantitative proteomic technique, comparing 40 patients with PSP to 40 with PD and 40 healthy controls [94]. The study identified 3653 proteins, revealing 190 proteins differentially expressed in PSP compared to the controls and 152 in PSP compared to PD [94]. Notably, a reduction in ATP6AP2 in the PSP patients demonstrated the highest diagnostic accuracy, with an area under the curve (AUC) of 0.922, followed by NEFM, EFEMP2, LAMP2, CHST12, FAT2, B4GALT1, LCAT, CBLN3, FSTL5, ATP6AP1, and GGH [94]. These altered proteins are primarily implicated in cell adhesion, glycan synthesis, and cholesterol metabolism [94].

Wise and co-authors conducted an unbiased DNA aptamer proteomics analysis, a method for identifying proteins based on their binding to specific DNA molecules, across three cohorts: originally diagnosed PSP-RS patients versus healthy controls, validated PSP-RS patients versus healthy controls, and PSP autopsy-confirmed cases versus FTLD confirmed without PSP patients [95]. The study employed enrichment and weighted consensus gene co-expression analyses, receiver-operating characteristic curves to assess diagnostic value, and linear regressions to determine associations with disease severity [95]. In the original cohort, 155 proteins were differentially expressed in PSP compared to the controls. Proteins associated with synaptic function, vesicle cytoskeletal trafficking, and cytokine–cytokine receptor interaction were most affected [95]. Notably, axon guidance was the most dysregulated pathway across all three cohorts. A panel of axon guidance proteins effectively discriminated between PSP and the controls, with very high accuracy (AUC 0.924, 0.815, and 0.932 in each cohort, respectively) [95]. Furthermore, two inflammatory proteins, i.e., galectin-10 and cytotoxic T lymphocyte-associated protein-4, showed a significant correlation with motor disability in the PSP-RS patients [95]. Notably, a high plasma/CSF ratio indicates significant contamination, which may skew the identified protein profiles and compromise the accuracy of biomarker studies [96].

### 2.7. Other Biomarkers

A wide spectrum of other potential biomarkers has been studied in PSP. Nonaka and co-authors examined CSF miRNA expression levels of 2632 miRNA arrays in 11 PSP patients, revealing significant dysregulation of miRNAs, particularly miR-204-3p, miR-873-3p, and miR-6840-5p, which target genes associated with the ubiquitin–proteasome system and autophagy pathways, critical processes implicated in neurodegenerative diseases like PSP [97].

Researchers have investigated neurotransmitter-based biomarkers in PSP and CBS; for example, Cerroni and co-authors reported significantly higher levels of noradrenaline in the CSF of PSP patients compared to those with PD [98]. A mass spectrometry study quantified 15 synaptic proteins in the CSF of PD, PSP, CBD, MSA, AD, and controls [99]. Neuronal pentraxin levels were reduced in PSP, PD, and MSA compared to the controls. Neurogranin, AP2B1, and complexin-2 were lower in PSP and MSA compared to the controls. Conversely, 14-3-3 zeta/delta, beta-synuclein, and gamma-synuclein were elevated in AD relative to parkinsonian disorders [99]. Kaiserova and co-authors investigated 5-hydroxyindoleacetic acid, the main serotonin metabolite, in the CSF of patients with PSP, CBS, PD, and MSA and the CSF of controls [100]. They observed reduced levels of the metabolite in the PD and MSA patients compared to the controls but no significant difference in the PSP and CBS patients [100].

Wellington and co-authors examined CSF neurogranin in neurodegenerative disorders and cognitively impaired individuals [101]. Neurogranin, a postsynaptic protein modulating calmodulin and synaptic plasticity, was significantly elevated in AD patients compared to PSP, PD, DLB, bvFTD, and PPA patients and a non-neurodegenerative cognitively impaired group. CSF neurogranin in AD correlated with t-tau and p-tau [101]. Hall and co-authors subsequently examined CSF neurogranin in PSP, CBS, PD, MSA, DLB, AD, and controls, confirming significantly elevated levels in AD compared to all other groups [102].

CSF orexin levels were examined in PSP, CBD, PD, and DLB. [103] Orexin was significantly lower in PSP and CBD than in PD and DLB. Notably, in PSP, orexin levels correlated with both disease duration and morbidity [103]. Given the implication of iron metabolism genes in tau-related neurodegeneration, Akiyama and co-authors examined CSF ferritin and transferrin in PSP, PD, and MSA [104]. They found that transferrin levels and transferrin-to-ferritin ratios were higher in PSP than in PD and MSA [104]. CSF Secernin-1, a protein involved in neurofibrillary tangle engagement in AD but not tau accumulation in primary tauopathies, shows increased levels in AD but not in PSP, CBD, PD, or MSA [105]. In contrast, CSF progranulin, implicated in FTLD spectrum diseases, exhibits no significant difference in levels across PSP, CBS, FTD, DLB, AD, and amnestic MCI [106]. Driven by the hypothesis that protein degradation contributes to neurodegeneration pathogenesis, Boman and co-authors investigated CSF levels of lysosomal proteins in clinically and pathologically defined PSP, CBS, CBD, and PD [107]. The study revealed decreased early endosomal antigen 1 and increased lysozyme in PSP, increased lysosomal-associated membrane proteins 1 and 2, microtubule-associated protein 1 light chain 3, and lysozyme in CBD, and decreased lysosomal-associated membrane proteins 1 and 2 in PD [107].

### 2.8. Cerebrospinal Fluid Biomarker Combinations

Table 3 summarizes the main findings of CSF biomarker combination studies.

Biomarker studies have explored combining markers to improve diagnostic accuracy for PSP and CBS. For example, Locascio and co-authors compared CSF t-tau and α-synuclein (ELISA) alongside serum α-synuclein in PSP, PD, AD, DLB, MSA, and NPH patients. [108]. The study showed limited discriminatory accuracy of CSF α-synuclein for PD/DLB vs. PSP/NPH (AUC 0.711) [108]. Compta and co-authors used RT-QuIC to measure CSF α-synuclein levels, in conjunction with CSF NfL and the midbrain-to-pons ratio, in patients with PSP, CBD, PD, and MSA and in healthy controls [109]. The combined biomarker panel demonstrated high diagnostic accuracy, with an AUC of 0.983 for distinguishing PD from APDs and an AUC of 0.924 for differentiating PSP and CBD from PD and MSA [109]. Konickova and co-authors investigated CSF concentrations of α-syn, t-tau, p-tau, Aβ42, neurofilament heavy chain and its phosphorylated form, clusterin, cystatin, and chromogranin A [110]. They enrolled patients with PSP, CBS, PD, DLB, and MSA and healthy controls. However, only the combined concentrations of the biomarkers in the CSF could provide some diagnostic validity in the differential diagnosis of MSA from PSP and CBD [110].

Constantinides and co-authors also examined CSF Aβ42, t-tau, p-tau181, and α-synuclein in a similar patient cohort (PSP, CBD, PD, MSA, and healthy controls) [111]. However, their analysis of specifically PSP and CBD yielded only one clinically relevant result: the CSF Aβ42/t-tau ratio could differentiate CBS patients from those with AD [111].

Alcolea and co-authors explored the diagnostic utility of combining NfL with AD biomarkers, specifically serum sAPPβ and YKL-40, in a cohort of patients with PSP, CBS, AD, bvFTD, and PPA and controls [112]. Their findings indicated that FTLD spectrum disorders had significantly lower sAPPβ levels than AD patients and the controls and that sAPPβ levels were associated with structural alterations in the frontal cortex and cingulum [112]. Furthermore, the ratios of sAPPβ/YKL-40 and NfL/sAPPβ achieved an AUC of 0.84 and 0.85 in differentiating FTLD spectrum disorders from AD, respectively [112]. In a study examining CSF NfL, p-tau, t-tau, and Aβ42 across PSP, CBS, AD, bvFTD, PPA, ALS, and controls, researchers found that ALS patients displayed higher NfL levels than PSP and CBS patients [113]. As anticipated, Aβ42 was lower in AD compared to FTLD groups. Notably, PSP patients had an increased p-tau/t-tau ratio relative to bvFTD, PPA, and ALS [113]. Meeter and co-authors also investigated CSF NfL and the p-tau/t-tau ratio across FTLD subtypes [114]. Their study included patients with PSP, CBS, bvFTD, PPA, and ALS and healthy controls. They found that NfL levels were highest and p-tau/t-tau ratios lowest in the ALS group, while the opposite pattern was observed in the logopenic variant of PPA [114]. However, the combined measurements of NfL and p-tau/t-tau did not effectively distinguish between FTLD-tau and FTLD-TDP pathologies [114]. Jepsson and co-authors measured CSF levels of Aβ peptides (Aβ42, Aβ40, Aβ38), MCP-1, p-tau, t-tau, sAPPα, sAPPβ, and NfL in patients with PSP, CBD, PD, MSA, AD, FTD, NPH, and VaD and healthy controls [115]. The patients with NPH exhibited lower t-tau and sAPP levels, along with elevated MCP-1, compared to the other neurodegenerative conditions [115].

Combined analysis of α-syn, AD-related biomarkers, and NfL is frequently investigated. Hall and co-authors examined α-syn, Aβ42, t-tau, and p-tau in PSP, PDD, DLB, MSA, AD, and healthy controls [116]. They observed significantly elevated NfL in APDs compared to PD [116]. In a prospective cohort study, Backstrom and co-authors enrolled participants with new-onset PSP, PD, and MSA and healthy controls, examining the same biomarker panel [117]. They found that the PD participants exhibited a distinct biomarker profile compared to PSP, with an area under the receiver operating characteristic curve (AUC) of 0.87 [117]. Magdalinou and co-authors examined CSF biomarkers, including α-syn, tau, Aβ, and NfL, alongside sAPPα, sAPPβ, and YKL-40, in PSP, CBS, PD, MSA, FTD, AD, and healthy controls [118]. Combined use of these nine biomarkers demonstrated high accuracy in discriminating PD from APDs (AUC 0.95) [118]. Specifically, PSP was differentiated from PD (AUC 0.95) and from MSA (AUC 0.84), while CBS was differentiated from PD (AUC 0.98) and from AD and FTD (AUC 0.98) [118]. In the only study focused on CSF biomarkers in PSP and CBS, Anastassiadis and co-authors found that 35.9% of CBS and 28.6% of PSP patients were α-synuclein-positive, as detected by SAA [119]. This positivity correlated with rapid eye movement sleep behavior disorder in both groups, and Aβ positivity correlated with age in α-synuclein-positive patients. The study also examined AD biomarkers and NfL [119].

Few studies have systematically reviewed CSF biomarkers for differentiating PSP and CBS from other neurodegenerative diseases. Xiang and co-authors conducted a meta-analysis to assess the diagnostic utility of CSF biomarkers in distinguishing PD from APDs and healthy controls [120]. The meta-analysis revealed that patients with PSP and CBS exhibited higher NfL levels compared to PD patients. Furthermore, PSP patients showed elevated Aβ concentrations relative to PD patients [120]. Remoli and co-authors’ systematic review of biomarkers for CBS suggests that NfL can be valuable for grouping patients in clinical trials involving CBS and other similar neurodegenerative diseases [121].

## 3. Discussion

Most CSF biomarker studies involving PSP and CBS were not specifically designed for these disorders. Instead, their primary objective was to distinguish PD from APDs, which incidentally include PSP and CBS. This design flaw often led to the aggregation of PSP and CBS data with other APDs, yielding ample comparative data between these disorders and PD. However, this approach resulted in a notable deficiency in data directly comparing PSP and CBS with other parkinsonian syndromes, such as MSA and DLB, hindering our ability to achieve nuanced differential diagnoses.

A similar issue arises when comparing PSP and CBS to other FTLD spectrum disorders, notably, bvFTD and PPA. The problem lies in the substantial clinical overlap between these syndromes. According to current diagnostic criteria, bvFTD and PPA can represent the clinical presentation of underlying PSP or CBS pathology [1,2]. Therefore, studies that group patients based on these clinical diagnoses may inadvertently compare groups with the same underlying neuropathological basis. This underscores the paramount importance of incorporating autopsy-confirmed pathology in study designs to ensure accurate group classifications and reliable research findings. Nevertheless, even autopsy-confirmed cases, while considered the gold standard, are not immune to potential selection bias [122]. These cases frequently derive from older cohorts recruited at specialized tertiary movement disorders clinics, which may not accurately represent the broader patient population [122].

An additional critical issue arises from the inherent design of the biomarkers used in PSP and CBS research. These biomarkers, including α-synuclein, a highly sensitive and specific marker for PD, and Aβ42 and p-tau217, crucial for AD diagnosis, are tailored to detect the pathological hallmarks of their respective diseases [12,13,69,123]. However, these proteins play a limited role in the underlying mechanisms of PSP and CBS [124]. As a result, their application in diagnosing PSP and CBS is largely indirect, functioning to rule out other relevant pathologies rather than directly confirming these specific disorders. Furthermore, it is essential to acknowledge that co-pathology is increasingly recognized as the norm in neurodegenerative disorders, with multiple pathologies often observed within an individual’s brain [125,126,127,128]. Consequently, the presence of α-synuclein or Aβ42 in PSP and CBS cases should be interpreted with caution [53,119]. These findings may reflect the presence of coexisting PD or AD pathology, rather than a direct indication of PSP- or CBS-specific biomarkers.

Moreover, although NfL demonstrates pathological elevation in both PSP and CBS, research often positions these disorders within a heterogeneous, intermediate group. This group lies between the significantly elevated NfL levels characteristic of ALS and the comparatively minor elevations found in PD and AD when compared to age-matched, healthy controls.

Furthermore, while tau biomarkers show promise in distinguishing between primary 4R and 3R tauopathies [28], data remain limited regarding their ability to differentiate between specific 4R tauopathies, such as PSP and CBD [28,29]. Critically, the biomarker profiles of PSP and CBS subtypes have received limited attention [24], despite the recognized potential for these subtypes to diverge in clinical course and progression [129]. Astonishingly, a solitary study group attempted to directly compare CSF biomarker profiles between PSP and CBS [53,119]. The vast majority of research either aggregates these disorders into a single group or fails to discover biomarkers that can definitively differentiate them, prioritizing the discrimination of these disorders from other neurodegenerative diseases through shared biomarker patterns. However, accurate differential diagnosis between PSP and CBS is particularly challenging due to their substantial clinical overlap, which may result in a significant number of patients being misdiagnosed. Autopsy confirmation often reveals that patients clinically diagnosed with CBS actually had PSP pathology, or the reverse [2,130]. The clinical overlap between PSP and CBS poses a significant threat to the reliability of future trials for therapies targeting these currently untreatable conditions, as misdiagnosis could lead to the inclusion of CBS patients in PSP treatment groups, or conversely.

Regardless, all of these studies fail to deal with the elephant in the room. The reason that no specific biomarkers for either PSP or CBS are studied is exactly because there are not any. Only two studies focus on MAPT and progranulin [34,106], which tend to be more commonly associated with PSP and CBD pathogenesis [131], and even they fail to present robust results.

A key obstacle lies in the diverse methodologies employed across studies. Discrepancies in analytical techniques, even when measuring the same biomarkers, create significant challenges for accurate interpretation. Different studies may use varying assay platforms, cutoff values, and sample processing protocols, all of which can yield divergent biomarker concentrations. As a result, direct comparison of absolute biomarker levels between studies becomes problematic, limiting the ability to draw consistent conclusions. This variability not only hampers statistical analyses but also renders meta-analyses potentially unreliable, as heterogeneity in methodology may introduce bias or mask true associations. Moreover, the interpretation of biomarker thresholds—which are critical for clinical decision-making—becomes ambiguous when studies report conflicting reference ranges or diagnostic criteria. Inconsistent and often contradictory findings stemming from methodological diversity ultimately undermine the standardization and reproducibility necessary for the clinical validation of reliable diagnostic biomarkers. To address this, there is a pressing need for harmonized protocols and consensus guidelines that would enable more meaningful comparison of results across independent cohorts and foster the development of universally applicable diagnostic algorithms. For example, while Borroni and co-authors reported a promising ability to differentiate PSP from other neurodegenerative diseases, including CBD, using a specific tau fragment ratio [25], subsequent attempts by another research group failed to replicate these findings [27].

Despite all the aforementioned challenges, several studies have yielded promising diagnostic results. Notably, the combined analysis of CSF α-synuclein, AD-related biomarkers, and NfL demonstrates high to very high diagnostic accuracy in differentiating PSP and CBS from PD, AD, MSA, and FTD [117,118]. Additionally, lysosomal protein profiling reveals distinct patterns differentiating PSP, CBD, and PD [107]. Proteomics analysis has identified several potential biomarkers with specificity for PSP, CBS, or both, including Wnt-1, ATP6AP2, and other targets implicated in glycan synthesis, cholesterol metabolism, and cell adhesion, potentially yielding valuable insights into the largely unknown pathogenesis of these multifactorial diseases [92,93]. Similarly, proteins associated with synaptic function, vesicle cytoskeletal trafficking, and cytokine–cytokine receptor interaction demonstrate high accuracy in discriminating PSP from other FTLD spectrum disorders [95]. However, as previously noted, the reproducibility of these findings requires further validation. Furthermore, the implementation of sophisticated biomarker analyses presents significant challenges for routine clinical practice and may pose substantial financial burdens for patients.

**Figure 1:**
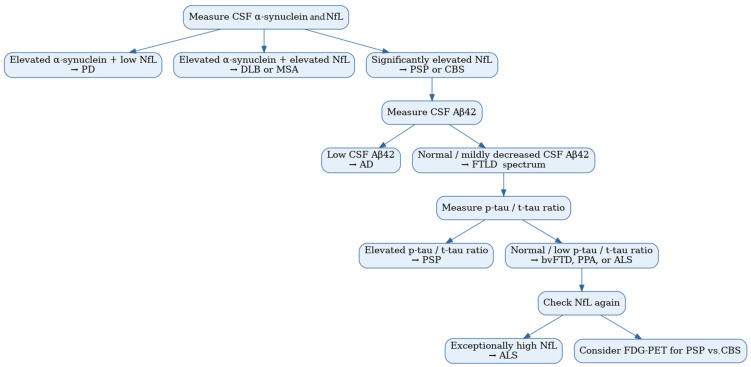
Proposed diagnostic flowchart utilizing CSF biomarkers in the diagnosis of PSP and CBS.

Nevertheless, based on the reviewed studies employing biomarker combinations, a stepwise approach could assist clinicians in the differential diagnosis of PSP and CBS from other neurodegenerative diseases. For example, given the high diagnostic accuracy of the combination of CSF α-synuclein and NfL, these two biomarkers could serve as an initial step to distinguish PSP and CBS from synucleinopathies such as PD, DLB, and MSA. Specifically, elevated CSF α-synuclein with lower NfL levels would favor a diagnosis of PD, while elevated levels of both biomarkers would be more indicative of DLB or MSA, effectively excluding synucleinopathies when not present [109,116]. In contrast, significantly elevated NfL levels would be more suggestive of PSP or CBS [62]. Based on the available data, we recommend the SIMOA technique as the most reliable method for measuring NfL levels and RT-QuIC for evaluating CSF α-synuclein. Subsequently, lower CSF Aβ42 levels could help differentiate AD from the FTLD spectrum [113]. Within the FTLD spectrum, the p-tau/t-tau ratio could then be applied, where elevated values may point towards PSP rather than other FTLD-related disorders, such as bvFTD, PPA, or ALS [113]. We suggest utilizing p-tau217 and p-tau181 levels, as these biomarkers are the most extensively studied in relation to PSP and CBS [30,34]. Their consistent use in recent research supports their reliability in the diagnostic process. Moreover, exceptionally high NfL levels may be more indicative of ALS than of PSP or CBS [114]. However, it is important to note that as the only study directly comparing PSP and CBS did not yield diagnostically significant results, no biomarker-based recommendation can currently be made for differentiating between PSP and CBS [16]. At this stage, other diagnostic techniques, such as FDG-PET, may prove helpful. A proposed diagnostic flowchart utilizing CSF biomarkers in PSP and CBS is demonstrated in Figure 1.

Regarding the utility of CSF biomarkers in the differential diagnosis between the various PSP and CBS subtypes, current guidance remains even more limited and ambiguous. To date, only one study, conducted by Srulijes and colleagues, has explored the role of CSF t-tau and p-tau in differentiating PSP-RS from PSP-P [24]. However, the study failed to demonstrate significant diagnostic utility, leaving this area of research unresolved. This lack of evidence highlights a crucial gap, as distinguishing between PSP and CBS subtypes is clinically important due to their differing prognoses and management strategies. Given the heterogeneity in PSP and CBS syndromes [1,2], further systematic investigation into subtype-specific biomarker profiles is urgently needed. Expanding research efforts in this direction could provide valuable insights and ultimately contribute to improving the accuracy of differential diagnosis in these complex and overlapping conditions.

Lastly, beyond their diagnostic utility, CSF biomarkers also contribute to a deeper understanding of the pathobiological processes underlying PSP and CBS. For example, increased CSF t-tau and p-tau concentrations reflect the core pathology of these disorders, both of which are primary 4R tauopathies [113]. Additionally, reduced Aβ42 concentrations and elevated α-synuclein levels suggest the presence of multiple coexisting pathologies in these patients [113,119]. The role of these pathologies in disease progression and their potential interactions with tau pathology remain to be determined. Moreover, significantly elevated NfL levels indicate extensive myelinated axon destruction in PSP and CBS, distinguishing them from other neurodegenerative diseases [62]. Inflammatory biomarkers further suggest a role for neuroinflammation in the pathogenesis of these disorders [79], while miRNAs provide valuable insights into altered gene expression within the brain [97]. Other biomarkers, such as Wnt-1—implicated in processes like cell adhesion and glycan synthesis—may help elucidate how PSP and CBS progress and spread within the brain [93]. Further investigation into the role of CSF biomarkers in understanding the pathogenesis of PSP and CBS is warranted.

## 4. Conclusions

In conclusion, CSF biomarkers could improve the differential diagnosis of PSP and CBS. The most promising results derive from a combination of biomarkers associated with PD, AD, and NfL. Furthermore, CSF proteomics analysis offers valuable insights into the pathogenesis of PSP and CBS and could also contribute to accurate diagnosis. However, research should focus specifically on these conditions, rather than using them as comparisons for other neurodegenerative diseases. Moreover, large-scale, multicenter cohort studies employing standardized methodologies could enhance the validity and reproducibility of the results. Given the frequent overlap in pathology between PSP and CBS, pathology-confirmed study cohorts should be used. Hopefully, these implementations will lead to improved diagnostic accuracy and understanding of these complex diseases, paving the way for effective treatments.

## Figures and Tables

**Table 1:** Studies analyzing CSF tau protein levels in PSP and/or CBS patients.

Study	4R ^1^ Tauopathy	Comparison Groups	CSF Biomarker	Main Findings
Srulijes and co-authors, 2011 [24]	PSP ^2^ (PSP-RS ^3^, PSP-P ^4^)	-	t-tau ^5^, p-tau ^6^	- t-tau and p-tau within normal range among both subtypes.
Borroni and co-authors, 2009 [25]	PSP	CBD ^7^, PD ^8^, DLB ^9^, AD ^10^, FTD ^11^, NCs ^12^	33kDa/55kDa tau ratio	- Reduced ratio in PSP compared to all other study groups.
Borroni and co-authors, 2010 [26]	PSP	CBD, PD, DLB, AD, FTD, HC	33kDa/55kDa tau ratio and midbrain-to-pons atrophy	- High sensitivity and moderate specificity in differentiating PSP from CBD and FTD.
Kuiperij and co-authors, 2012 [27]	PSP	-	33kDa/55kDa tau ratio	- Undetectable levels of 33 kDa and 55 kDa tau fragments.
Heikkinen and co-authors, 2025 [8]	PSP, CBS ^13^	bvFTD ^14^	t-tau, p-tau	- Lower t-tau and p-tau in PSP compared to bvFTD.
Saijo and co-authors, 2017 [28]	PSP, CBD	Pick’s disease, AD, DLB, bvFTD, ALS ^15^, FTLD-TDP ^16^, FTLD-17 ^17^, CTE ^18^, CIDP ^19^, cerebrovascular disease	3R ^20^ tau fragment	- High sensitivity and specificity in differentiating 3R-related Pick’s disease from PSP and CBD.
Saijo and co-authors, 2020 [29]	PSP, CBD	Pick’s disease, DLB, MSA, ALS, FTLD-TDP, FTLD-17, CTE, PART ^21^	4R tau fragment	- Samples from living patients exhibited weak seeding activities. - Assay responses were significantly higher in patients with clinically diagnosed PSP or CBS.
Wagshal and co-authors, 2015 [30]	PSP	AD, NCs	t-tau, p-tau181	- Higher t-tau and p-tau181 in AD compared to PSP and NCs. - Lower N-terminal and C-terminal tau for PSP than AD and NCs.
Cicognola and co-authors, 2021 [31]	PSP	PD, AD, MSA, MCI ^22^, SCD ^23^	N-224 tau fragment	- Higher N-224 tau fragment in AD compared to all other study groups.
Horie and co-authors, 2022 [32]	PSP, CBD	AD, Pick’s disease, AGD ^24^	Two 4R tau species with MTBDs ^25^	- Lower 4R tau species in AD compared to all other study groups.
Dilcher and co-authors, 2024 [33]	PSP, CBS	AD	p-tau181	- Increased p-tau181 in AD compared to PSP and CBS.
Sato and co-authors, 2021 [34]	PSP, CBS	AD, bvFTD	p-tau217/t-tau217 ratio, Aβ42/Aβ40 ^26^ ratio	- High p-tau217/t-tau217 ratio and low Aβ42/Aβ40 ratio in AD. - Low p-tau217/t-tau217 ratio and normal Aβ42/Aβ40 ratio in PSP, CBS, and bvFTD.

^1^ 4R: four-repeat, ^2^ PSP: progressive supranuclear palsy, ^3^ PSP-RS: PSP-Richardson’s syndrome, ^4^ PSP-P: PSP with predominant parkinsonism, ^5^ t-tau: total tau, ^6^ p-tau: hyperphosphorylated tau, ^7^ CBD: corticobasal degeneration, ^8^ PD: Parkinson’s disease, ^9^ DLB: dementia with Lewy bodies, ^10^ AD: Alzheimer’s disease, ^11^ FTD: frontotemporal dementia, ^12^ NCs: normal controls, ^13^ CBS: corticobasal syndrome, ^14^ behavioral variant FTD, ^15^ ALS: amyotrophic lateral sclerosis, ^16^ FTLD-TDP, frontotemporal lobar degeneration with TDP-43, ^17^ FTLD-17: frontotemporal dementia with parkinsonism linked to chromosome 17, ^18^ CTE: chronic traumatic encephalopathy, ^19^ CIDP: chronic inflammatory demyelinating polyneuropathy, ^20^ 3R-tau, ^21^ PART: primary age-related tauopathy, ^22^ MCI: mild cognitive impairment, ^23^ SCD: subjective cognitive decline, ^24^ AGD: argyrophilic grain disease, ^25^ MTBD: microtubule binding domains, ^26^ Aβ: amyloid beta.

**Table 2:** Studies analyzing CSF NfL levels in PSP and/or CBS patients.

Study	4R ^1^ Tauopathy	Comparison Groups	CSF Biomarker	Main Findings
Holmberg and co-authors, 1998 [57]	PSP ^2^	PD ^3^, MSA ^4^	NfL ^5^	- Elevated NfL and NfH in PSP and MSA compared to PD.
Brettschneider and co-authors, 2006 [58]	PSP, CBD ^6^	PD, MSA	NfH ^7^	- High specificity and moderate sensitivity for differentiating PSP from PD.
Painous and co-authors, 2023 [59]	PSP, CBD	PD, MSA, NCs ^8^	NfL	- Higher NfL in PSP, CBD, and MSA compared to PD and NCs.
Oliveira Hauer and co-authors, 2023 [60]	PSP	PD, DLB ^9^, NCs	NfL	- NfL could separate PSP from PD, DLB, and NCs.
Scherling and co-authors, 2014 [61]	PSP, CBS ^10^	AD ^11^, PD, bvFTD ^12^, PPA ^13^, NCs	NfL	- Elevated NfL in bvFTD and PPA compared to all other groups.
Olsson and co-authors, 2019 [62]	PSP, CBS	PD, PD with MCI ^14^, PDD ^15^, DLB, FTD, ALS ^16^, AD, MCI, NCs	NfL	- Highest NfL in ALS and FTD. - Highest NfL in PSP and CBS among parkinsonian syndromes.
Ge and co-authors, 2018 [63]	PSP, CBD	PD, MSA	NfL	- Moderate sensitivity and specificity in differentiating PD from other diseases.
Wang and co-authors, 2019 [64]	PSP, CBD	PD, MSA, ALS, CJD ^17^, HD ^18^	NfL	- Highest NfL elevation in ALS, CJD, and HD. - NfL levels within normal range in PD. - Significant elevations in PSP and MSA.

^1^ 4R: four-repeat. ^2^ PSP: progressive supranuclear palsy ^3^ PD: Parkinson’s disease, ^4^ MSA: multiple system atrophy, ^5^ NfL: neurofilament light chain, ^6^ CBD: corticobasal degeneration, ^7^ NfH: neurofilament heavy chain, ^8^ NCs: normal controls, ^9^ DLB: dementia with Lewy bodies, ^10^ CBS: corticobasal syndrome, ^11^ AD: Alzheimer’s disease, ^12^ bvFTD: behavioral variant frontotemporal dementia, ^13^ PPA: primary progressive aphasia, ^14^ MCI: mild cognitive impairment, ^15^ PDD: PD dementia, ^16^ ALS: amyotrophic lateral sclerosis ^17^ CJD: Creutzfeldt Jakob disease, ^18^ HD: Huntington’s disease.

**Table 3:** Studies analyzing CSF biomarker combinations in PSP and/or CBS patients.

Study	4R ^1^ Tauopathy	Comparison Groups	CSF Biomarker	Main Findings
Locascio and co-authors, 2011 [108]	PSP ^2^	PD ^3^, AD ^4^, DLB ^5^, MSA ^6^, NPH ^7^	t-tau ^8^, α-syn ^9^	- Limited discriminatory accuracy of α-syn for PD/DLB vs. PSP/NPH.
Compta and co-authors, 2022 [109]	PSP, CBD ^10^	PD, MSA, NCs ^11^	α-syn, NfL ^12^	- High accuracy for differentiating PSP and CBD from PD and MSA.
Konickova and co-authors, 2022 [110]	PSP, CBS ^13^	PD, DLB, MSA, NCs	t-tau, p-tau ^14^, Aβ42 ^15^, NfH ^16^, clusterin, cystatin, chromogranin A	- Minimum diagnostic validity for combined biomarker concentrations in the differential diagnosis of MSA from PSP and CBD.
Constantinides and co-authors, 2017 [111]	PSP, CBD	PD, MSA, NCs	t-tau, p-tau181, Aβ42, α-syn	- Aβ42/t-tau ratio could differentiate CBS from AD patients.
Alcolea and co-authors, 2017 [112]	PSP, CBS	AD, bvFTD ^17^, PPA ^18^, NCs	NfL, sAPPβ ^19^, YKL-40	- Lower sAPPβ in PSP, CBS, bvFTD, and PPA than in AD and NCs. - Medium efficacy of sAPPβ/YKL-40 and NfL/sAPPβ ratios in differentiating other diseases from AD.
Abu-Rumeileh and co-authors 2018 [113]	PSP, CBS	AD, bvFTD, PPA, ALS ^20^, NCs	t-tau, p-tau, NfL, Aβ42	- Lower Aβ42 in AD compared to FTLD ^21^ groups. - Increased p-tau/t-tau ratio in PSP compared to bvFTD, PPA, and ALS.
Meeter and co-authors, 2018 [114]	PSP, CBS	bvFTD, PPA, ALS, NCs	t-tau, p-tau, NfL	- Highest NfL levels and lowest p-tau/t-tau ratio in an ALS group. - Opposite pattern in the logopenic variant of PPA.
Jeppsson and co-authors, 2019 [115]	PSP, CBD	PSP, CBD, PD, MSA, AD, FTD, NPH, VaD ^22^	Aβ42, Aβ40, Aβ38, MCP-1 ^23^, p-tau, t-tau, sAPPα, sAPPβ, NfL	- Lower t-tau and sAPP and elevated MCP-1 for NPH compared to other disease groups.
Hall and co-authors, 2012 [116]	PSP	PDD ^24^, DLB, MSA, AD, and healthy controls	α-syn, Aβ42, t-tau, p-tau	- Elevated NfL in APDs ^25^ compared to PD.
Backstrom and co-authors, 2015 [117]	PSP	PD, MSA, NCs	α-syn, Aβ42, t-tau, p-tau	- Distinct biomarker profile in PD compared to PSP.
Magdalinou and co-authors, 2015 [118]	PSP, CBS	PD, MSA, FTD, AD, NCs	α-syn, tau, Aβ, NfL, sAPPα, sAPPβ, YKL-40	- PSP was differentiated from PD (AUC 0.95) and from MSA (AUC 0.84). - CBS was differentiated from PD (AUC 0.98) and from AD and FTD (AUC 0.98).
Anastassiadis and co-authors, 2024 [119]	PSP, CBS	-	α-syn, Aβ42, p-tau, t-tau, NfL	- Overall, 35.9% of CBS and 28.6% of PSP patients were α-syn-positive. - Aβ positivity correlated with age in α-synuclein-positive patients.

^1^ 4R: four-repeat. ^2^ PSP: progressive supranuclear palsy ^3^ PD: Parkinson’s disease, ^4^ AD: Alzheimer’s disease, ^5^ DLB: dementia with Lewy bodies, ^6^ MSA: multiple system atrophy, ^7^ NPH: normal pressure hydrocephalus, ^8^ t-tau: total tau, ^9^ α-syn: α-synuclein, ^10^ CBD: corticobasal degeneration, ^11^ NCs: normal controls, ^12^ NfL: neurofilament light chain, ^13^ CBS: corticobasal syndrome, ^14^ p-tau: hyperphosphorylated tau, ^15^ Aβ: amyloid beta, ^16^ NfH: neurofilament heavy chain, ^17^ bvFTD: behavioral variant frontotemporal dementia, ^18^ PPA: primary progressive aphasia, ^19^ sAPP: soluble amyloid precursor protein, ^20^ ALS: amyotrophic lateral sclerosis, ^21^ FTLD: frontotemporal lobar degeneration, ^22^ VaD: vascular dementia, ^23^ MCP-1: Monocyte chemotactic protein-1, ^24^ PDD: PD dementia, ^25^ APD: atypical parkinsonian disorder.

## Data Availability

No new data were created during the preparation of this manuscript.

## Abbreviations

The following abbreviations are used in this manuscript:

3R	three-repeat
4R	four-repeat
Aβ	amyloid beta
AD	Alzheimer’s disease
AGD	argyrophilic grain disease
ALS	amyotrophic lateral sclerosis
APD	atypical parkinsonian disorder
APP	amyloid precursor protein
AUC	area under the curve
bvFTD	behavioral variant of frontotemporal dementia
CBD	corticobasal degeneration
CBS	corticobasal syndrome
CHIT1	chitotriosidase 1
CNS	central nervous system
CSF	cerebrospinal fluid
CTE	chronic traumatic encephalopathy
DaT	dopamine transporter
DLB	dementia with Lewy bodies
ELISA	enzyme-linked immunosorbent assay
FDG	fludeoxyglucose-18
FTD	frontotemporal dementia
FTLD	frontotemporal lobar degeneration
FTLD-TDP	frontotemporal lobar degeneration with TDP 43
FTLD-17	frontotemporal dementia with parkinsonism linked to chromosome 17
GFAP	glial fibrillary acidic protein
IL-1β	interleukin 1β
IL-6	interleukin 6
MAPT	microtubule-associated protein Tau gene
MCI	mild cognitive impairment
MCP-1	monocyte chemotactic protein-1
MRI	magnetic resonance imaging
MSA	multiple system atrophy
MTBDs	microtubule-binding domains
NfL	neurofilament light chain
NPH	normal pressure hydrocephalus
PAR	protein aggregation rate
PCA	posterior cortical atrophy
PD	Parkinson’s disease
PDD	Parkinson’s disease dementia
PET	positron emission tomography
PPA	primary progressive aphasia
PSP	progressive supranuclear palsy
PSP-P	PSP-Richardson’s syndrome
PSP-RS	PSP with predominant parkinsonism
p-tau	hyperphosphorylated tau
p-tau181	tau phosphorylated at threonine 181
p-tau217	tau phosphorylated at threonine 217
RT-QuIC	real-time quaking-induced conversion
SAA	seed amplification assay
sAPP	soluble amyloid precursor protein
SCD	subjective cognitive decline
SPECT	single-photon emission computed tomography
TNF-α	tumor necrosis factor α
t-tau	total tau
UCH-L1	ubiquitin carboxy-terminal hydrolase L1
VaD	vascular dementia
VBM	voxel-based morphometry
YKL-40	chitinase-3-like-1
